# Proteomic analysis of SETD6 interacting proteins

**DOI:** 10.1016/j.dib.2016.01.042

**Published:** 2016-01-29

**Authors:** Ofir Cohn, Ayelet Chen, Michal Feldman, Dan Levy

**Affiliations:** a The Shraga Segal Department of Microbiology, Immunology and Genetics, Israel; b The National Institute for Biotechnology in the Negev, Ben-Gurion University of the Negev, P.O.B. 653, Be׳er-Sheva, 84105 Israel

**Keywords:** SETD6, Mass spectrometry, Interactome

## Abstract

SETD6 (SET-domain-containing protein 6) is a mono-methyltransferase that has been shown to methylate RelA and H2AZ. Using a proteomic approach we recently identified several new SETD6 substrates. To identify novel SETD6 interacting proteins, SETD6 was immunoprecipitated (IP) from Human erythromyeloblastoid leukemia K562 cells. SETD6 binding proteins were subjected to mass-spectrometry analysis resulting in 115 new SETD6 binding candidates. STRING database was used to map the SETD6 interactome network. Network enrichment analysis of biological processes with Gene Ontology (GO) database, identified three major groups; metabolic processes, muscle contraction and protein folding.

## **Specifications Table**

**Table:** 

Subject area	*Biology*
More specific subject area	Human SETD6 binding proteins
Type of data	Figures
How data was acquired	Immunoprecipitation, mass spectrometry, STRING and GO bioinformatics analysis
Data format	Analyzed
Experimental factors	Please see details in the experimental design section below
Experimental features	Endogenous SETD6 protein from K562 cells was immunoprecipitated with monoclonal SETD6 antibody conjugated to beads or beads only as a control. SETD6-bound proteins were then separated by SDS-PAGE and stained with coomassie. Gels were sliced to 1mm squares and subjected to mass-spectrometry.
Data source location	Department of Microbiology, Immunology and Genetics and the National institute for biotechnology in the Negev, Ben-Gurion University of the Negev, P.O.B. 653, Be׳er-Sheva 84105, Israel
Data accessibility	Supported data can be obtained from Chen et. al, (http://dx.doi.org/10.1016/j.bbagrm.2016.01.003

## **Value of the data**

• Our results reveal new proteins that interact with SETD6. Research can utilize this data to investigate SETD6 biology and its link to their protein of interest.
• This data links SETD6 to the regulation of 3 defined processes, all of which are involved in the pathophysiology of several human diseases and has therefore the potential of opening new research directions.
• The information presented in this study could be used by researchers to identify new SETD6 substrates.

## 1. Data

SETD6-bound proteins were immunoprecipitated by SETD6 antibody and subjected to mass-spectrometry analysis results in the identification of 115 new SETD6 cellular interacting proteins candidates [1].

**Fig. 1:**
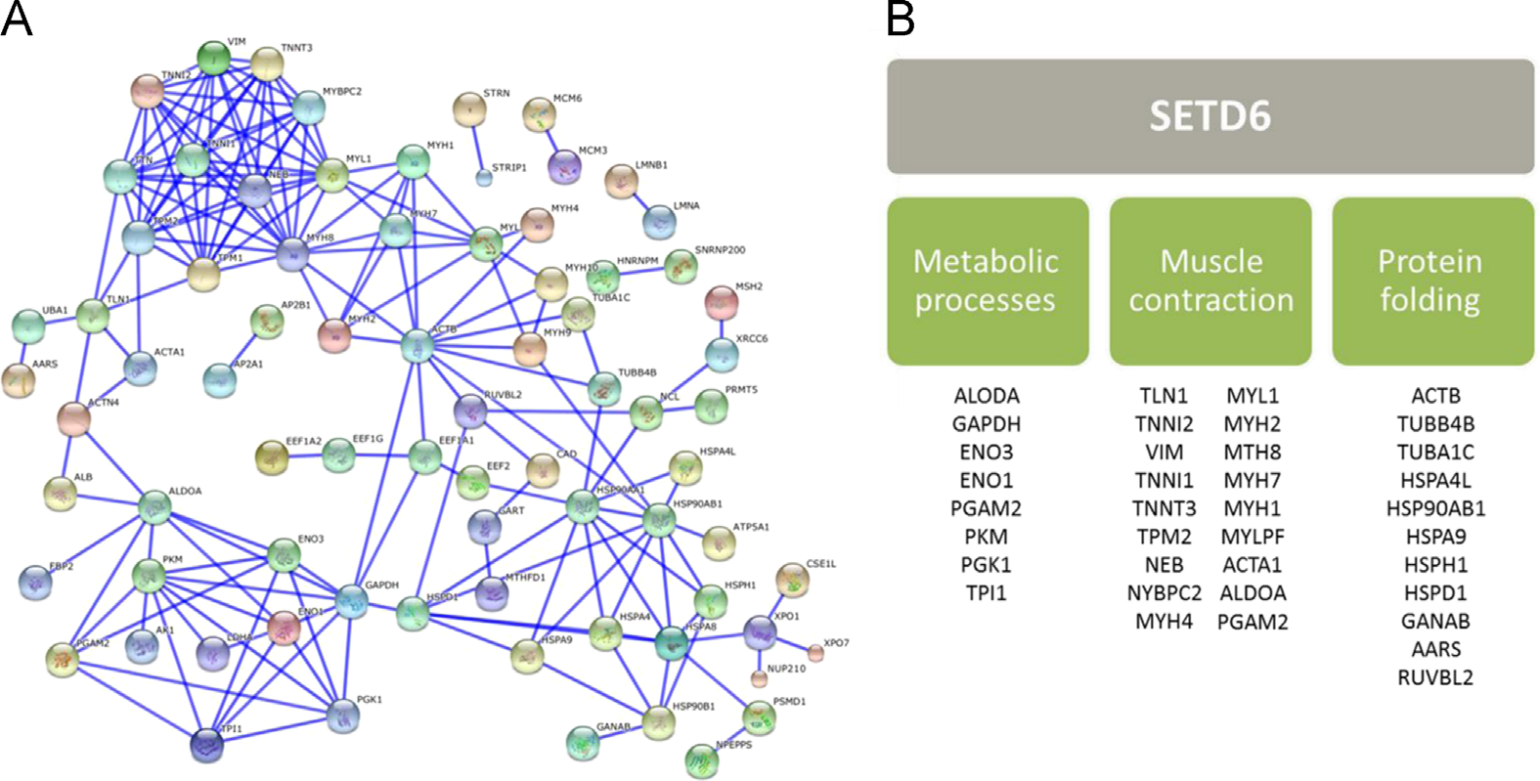
Mass-spectrometry analysis: (A) Protein–protein interactions from the STRING interaction database. Proteins are represented as nodes. (B) Classification of proteins based on Gene Ontology (GO) biological processes.

These proteins were then further analyzed by STRING database [2] to create SETD6 interacting proteins network (Fig.1A). Disconnected nodes and low *p*-values interactions were filtered out. Enrichment of Gene Ontology (GO) biological processes analysis [3], [4] was then utilized to classify the newly identified proteins into three major groups; metabolic processes, muscle contraction, and protein folding (Fig. 1B).

## 2. Experimental design, materials and methods

### 2.1. Immunoprecipitation

Cells were lysed in RIPA lysis buffer (50 mM Tris–HCl, pH 8, 150 mM NaCl, 1% Nonidet P-40, 0.5% deoxycholate, 0.1% SDS (v/v), 1 mM dithiothreitol (DTT) and Sigma protease inhibitor cocktail (P8340 diluted 1:100)). Lysates were incubated for 1 h at 4 °C with 10 ul protein A/G beads (Santa Cruz Biotechnology) as a pre-clear step. Pre-cleared lysates were incubated overnight at 4 °C with SETD6 antibody (1 ug) conjugated to beads or beads only as control. After incubation, beads were washed 4 times with lysis buffer, heated at 95 °C for 5 min in Laemmli sample buffer, and resolved by SDS-PAGE.

### 2.2. Mass-spectrometry

SETD6- bound proteins were immunoprecipitated using SETD6 antibody conjugated to beads or beads only as a control. After SETD6 immunoprecipitation, SETD6-bound proteins were separated by SDS-PAGE and stained with coomassie. Gels were sliced to 1mm squares and subjected to mass-spectrometry as previously described in Elharar et al. [5].

### 2.3. Bioinformatic analysis

For interpretation of MS/MS spectra we preformed STRING protein–protein interaction (string-db.org) with enrichment of Gene Ontology (GO) analysis of these data sets to predict which biological processes as well as interactive networks these genes are involved in.

**Conflict of interest**

The authors declare no conflict of interest.
