# Supplementary material for: Proteomic analysis of SETD6 interacting proteins
Source: Data Brief. 2016 Jan 29;6:799–802. doi: 10.1016/j.dib.2016.01.042 (PMC4749940; doi:10.1016/j.dib.2016.01.042)
Supplement: Supplementary file 1 — Supplementary material [file mmc1.doc]

**Conflict of interest**

The authors declare no conflict of interest.
